# Diagnostic approaches for tubal factor infertility: a systematic review of HSG, HyCoSy, HyFoSy, and laparoscopy

**DOI:** 10.3389/frph.2026.1861471

**Published:** 2026-10-07

**Authors:** Adalyat Alenova, Almagul Kauysheva, Anuar Korkan, Islam Magalov

**Affiliations:** 1 Kazakh Medical University “KSPH”, Almaty, Kazakhstan; 2 Department of Science and Human Resources, Ministry of Health of the Republic of Kazakhstan, Astana, Kazakhstan; 3 Department of Obstetrics and Gynecology № 1, Medical University of Astana, Astana, Kazakhstan; 4 Department of Obstetrics and Gynecology, Baku Branch of the First Moscow State Medical University, Baku, Azerbaijan

**Keywords:** diagnostic accuracy, hysterosalpingo-contrast sonography, hysterosalpingo-foam sonography, hysterosalpingography, laparoscopy, tubal infertility

## Abstract

**Introduction:**

Tubal pathology remains a major cause of female infertility, making accurate assessment of tubal patency and morphology important for clinical decision-making. Several diagnostic approaches are available, including hysterosalpingography (HSG), hysterosalpingo-contrast sonography (HyCoSy), hysterosalpingo-foam sonography (HyFoSy), and laparoscopy with chromopertubation. This systematic review aimed to synthesize current evidence on their diagnostic performance, clinical applicability, and role in contemporary diagnostic pathways.

**Methods:**

PubMed/MEDLINE, Embase, the Cochrane Library, and Web of Science were searched for studies published between January 2015 and March 2026. Studies evaluating methods for tubal patency or morphological assessment in women undergoing infertility investigation were considered. A total of 21 studies were included. Owing to clinical and methodological heterogeneity, particularly differences in study populations, diagnostic thresholds, and reference standards, the findings were synthesized qualitatively.

**Results:**

HSG generally demonstrated high sensitivity but variable specificity, limiting its reliability for confirming tubal occlusion. HyCoSy showed potentially useful diagnostic performance as a non-invasive screening method, although estimates varied across studies. Several studies reported favorable specificity, predictive values, and agreement measures for HyFoSy, together with improved patient tolerability; however, findings were not uniform and were influenced by methodological heterogeneity and differences in reference standards. Laparoscopy with chromopertubation remained the reference standard for assessing tubal patency and identifying concomitant pelvic pathology, although its invasive nature limits its routine use.

**Discussion:**

Current evidence supports a stepwise approach to tubal assessment in which less invasive methods may be used initially, with confirmatory or invasive techniques reserved for selected cases. Such an approach may improve diagnostic confidence while reducing unnecessary procedural burden and risks. Further well-designed comparative studies using standardized reference standards and outcome definitions are needed to clarify the relative diagnostic value of available techniques.

## 1 Introduction

Tubal pathology remains a major contributor to female infertility, accounting for approximately 30%–40% of cases worldwide (1, 2). Structural and functional abnormalities of the fallopian tubes, most commonly resulting from pelvic inflammatory disease, endometriosis, postoperative adhesions, or hydrosalpinx, can impair oocyte transport, fertilization, and embryo migration, ultimately reducing the likelihood of natural conception. Accurate evaluation of tubal patency and morphology is therefore a critical component of infertility assessment and directly influences clinical decision-making, including the choice between expectant management, surgical intervention, and assisted reproductive technologies (1, 3).

Over the past decades, a wide range of diagnostic approaches has been developed to assess tubal status. Hysterosalpingography (HSG) has traditionally been used as a first-line imaging modality because of its accessibility and relatively low cost, while laparoscopy with chromopertubation remains the reference standard for assessing tubal patency and simultaneously visualizing associated pelvic pathology (4, 5). More recently, less invasive techniques such as hysterosalpingo-contrast sonography (HyCoSy) and advanced ultrasound-based methods have gained increasing attention as potential alternatives, offering improved patient tolerability and reduced procedural risks (6). In addition, hysterosalpingo-foam sonography (HyFoSy), a newer ultrasound-based technique using foam contrast agents, has emerged as a promising minimally invasive option, with several studies reporting favorable diagnostic performance and improved patient comfort (7). At the same time, emerging approaches, including artificial intelligence-assisted imaging and molecular or immunohistochemical assessment, may further enhance diagnostic accuracy and enable more individualized evaluation of tubal function (8, 9).

Despite these advances, the optimal diagnostic strategy for tubal infertility remains unclear. Existing studies vary considerably in design, patient selection, and outcome definitions, leading to heterogeneity in reported diagnostic performance and limiting the ability to directly compare modalities (4, 7, 10, 11). Moreover, the choice of diagnostic method in clinical practice is often influenced not only by accuracy, but also by invasiveness, availability, cost, and patient characteristics (12). As a result, clinicians face challenges in selecting the most appropriate investigation for individual patients.

The aim of this systematic review is to synthesize current evidence on diagnostic approaches for fallopian tube assessment in women with infertility, with a particular focus on their clinical applicability, advantages, and limitations, and potential role within contemporary diagnostic pathways. By integrating findings across studies, this review seeks to provide a structured overview of existing methods and to support more informed, patient-centered decision-making in the evaluation of tubal factor infertility.

## 2 Methods

### 2.1 Study design and reporting standards

This systematic review was conducted in accordance with the PRISMA 2020 guidelines. The review protocol was not prospectively registered. Because of substantial clinical and methodological heterogeneity among the included studies, a quantitative meta-analysis was not performed. The included studies differed in index tests, contrast media, reference standards, diagnostic thresholds, units of analysis, outcome reporting, and risk of bias. In addition, some studies reported diagnostic-accuracy estimates against laparoscopy with chromopertubation, whereas others reported agreement or concordance between non-invasive tests. Therefore, a qualitative synthesis was considered more appropriate.

### 2.2 Search strategy

A comprehensive literature search was conducted in PubMed/MEDLINE, Embase, Cochrane Library, and Web of Science to identify relevant studies published between January 2015 and March 2026. The search strategy combined Medical Subject Headings (MeSH) and free-text terms related to tubal infertility and diagnostic methods, including “tubal infertility”, “fallopian tube obstruction”, “hysterosalpingography”, “hysterosalpingo-contrast sonography (HyCoSy)”, “hysterosalpingo-foam sonography (HyFoSy)”, “laparoscopy with chromopertubation”, “tubal patency”, and “diagnostic accuracy” (Supplementary Table S1). The search was performed independently by two reviewers and was supplemented by manual screening of reference lists of eligible studies to identify additional relevant publications.

### 2.3 Inclusion and exclusion criteria

Eligibility criteria were defined according to the PICOS framework:

Population (P): Women of reproductive age undergoing infertility evaluation in whom assessment of fallopian-tube patency or tubal-factor infertility was clinically indicated.

Index test/intervention (I): Diagnostic methods used to evaluate fallopian-tube patency or morphology, including hysterosalpingography (HSG), hysterosalpingo-contrast sonography (HyCoSy), hysterosalpingo-foam sonography (HyFoSy), and related imaging-based techniques.

Comparator/reference standard (C): Laparoscopy with chromopertubation, hysteroscopy combined with laparoscopy, or another clearly defined diagnostic method used for comparison of tubal-patency findings. Studies comparing two or more non-invasive diagnostic modalities were also eligible.

Outcomes (O): Studies were required to report at least one relevant diagnostic outcome, including sensitivity, specificity, diagnostic accuracy, positive predictive value (PPV), negative predictive value (NPV), likelihood ratios, agreement or concordance measures, or false-positive and false-negative findings. Patient-centered outcomes, including procedural pain, tolerability, adverse events, and clinical applicability, were extracted when available.

Study design (S): Eligible study designs included randomized controlled trials, prospective or retrospective cohort studies, cross-sectional diagnostic-accuracy studies, and comparative observational studies published as full-text articles in English between January 2015 and March 2026.

Studies were excluded if they focused primarily on non-tubal causes of infertility, such as isolated ovulatory disorders, endocrine disorders, uterine-factor infertility, or male-factor infertility, and did not report separate findings for tubal assessment. Studies in which tubal-patency outcomes could not be extracted separately were also excluded. Additional exclusion criteria were case reports, small non-comparative case series, conference abstracts, editorials, letters, narrative reviews, systematic reviews, animal or laboratory studies, non-English-language publications, and studies lacking sufficient information on diagnostic or patient-centered outcomes.

### 2.4 Study selection and data extraction

All identified records were imported into a reference management system, and duplicates were removed. Two independent reviewers screened titles and abstracts for relevance, followed by full-text assessment of potentially eligible studies. Disagreements were resolved through discussion and consensus.

Data were independently extracted by two reviewers using a standardized data-collection form. Extracted variables included study design, country, sample size, patient characteristics, diagnostic modality, contrast medium or foam formulation, reference standard, unit of analysis, and reported diagnostic outcomes, including sensitivity, specificity, accuracy, PPV, NPV, likelihood ratios, and agreement measures. Patient-centered and practical outcomes, including procedural pain, tolerability, adverse events, radiation exposure, outpatient feasibility, operator requirements, and clinical applicability, were also extracted when available.

### 2.5 Quality assessment

The methodological quality of the included studies was assessed using appraisal tools selected according to study design and analytical purpose. Diagnostic-accuracy studies comparing an index test with a defined reference standard were evaluated using the Quality Assessment of Diagnostic Accuracy Studies-2 (QUADAS-2) tool. Risk of bias was assessed across four domains: patient selection, index test, reference standard, and flow and timing. Applicability concerns were considered for the patient-selection, index-test, and reference-standard domains.

Studies primarily evaluating agreement or concordance between diagnostic methods without an independent reference standard were assessed using the Joanna Briggs Institute Critical Appraisal Checklist for Analytical Cross-Sectional Studies. This checklist evaluates the clarity of inclusion criteria, description of participants and setting, validity and reliability of measurements, identification and management of confounding factors, and appropriateness of the statistical analysis.

Two reviewers independently completed all quality assessments, and disagreements were resolved through discussion and consensus.

### 2.6 Qualitative synthesis

A quantitative meta-analysis was not performed because the included studies were not sufficiently homogeneous for meaningful statistical pooling. Studies differed in design, patient populations, prevalence of tubal pathology, index-test protocols, contrast media or formulations, reference standards, diagnostic thresholds, and units of analysis. In addition, outcome reporting was inconsistent: some studies reported sensitivity and specificity, whereas others reported accuracy, predictive values, likelihood ratios, agreement statistics, or concordance only. Because agreement between two non-invasive tests is not equivalent to diagnostic accuracy against an independent reference standard, pooling these estimates would have been methodologically inappropriate. Therefore, a qualitative synthesis was conducted.

Studies were grouped according to the index modality evaluated: HSG, HyCoSy, and HyFoSy. Laparoscopy with chromopertubation was considered primarily as the reference standard, while its clinical role, invasiveness, and ability to identify associated pelvic pathology were discussed separately.

Diagnostic outcomes were extracted using standardized categories, including sensitivity, specificity, overall diagnostic accuracy, positive predictive value (PPV), negative predictive value (NPV), likelihood ratios, and agreement statistics. Outcomes not provided by the original studies were recorded as not reported and were not inferred or recalculated unless sufficient raw data were available.

Safety, tolerability, and practical applicability were also synthesized qualitatively. These outcomes included procedural pain, patient acceptability, adverse events, radiation exposure, contrast-related considerations, outpatient feasibility, operator dependence, and equipment requirements.

Results were presented in structured tables containing standardized outcome columns. Diagnostic-accuracy findings obtained against laparoscopy with chromopertubation were distinguished from agreement or concordance findings obtained through comparison with another non-invasive test. No data imputations were performed, and all analyses were based on information reported in the original publications. Formal statistical heterogeneity and sensitivity analyses were not conducted because no quantitative synthesis was undertaken.

## 3 Results

### 3.1 Study selection

A total of 1,312 records were identified through database searches. After removing duplicates, 1,165 studies remained and were screened based on their titles and abstracts. Of these, 998 records were excluded at this stage. The full texts of 167 articles were then assessed for eligibility. Following full-text review, 146 articles were excluded. The reasons for exclusion were: not meeting the eligibility criteria or not reporting extractable tubal-patency outcomes (*n* = 109), full text unavailable despite attempted access (*n* = 25), and publication in a language other than English (*n* = 12). These exclusions are shown in the PRISMA flow diagram (Figure 1). In the end, 21 studies met the inclusion criteria and were included in this review. The study selection process is presented in Figure 1.

**Figure 1:**
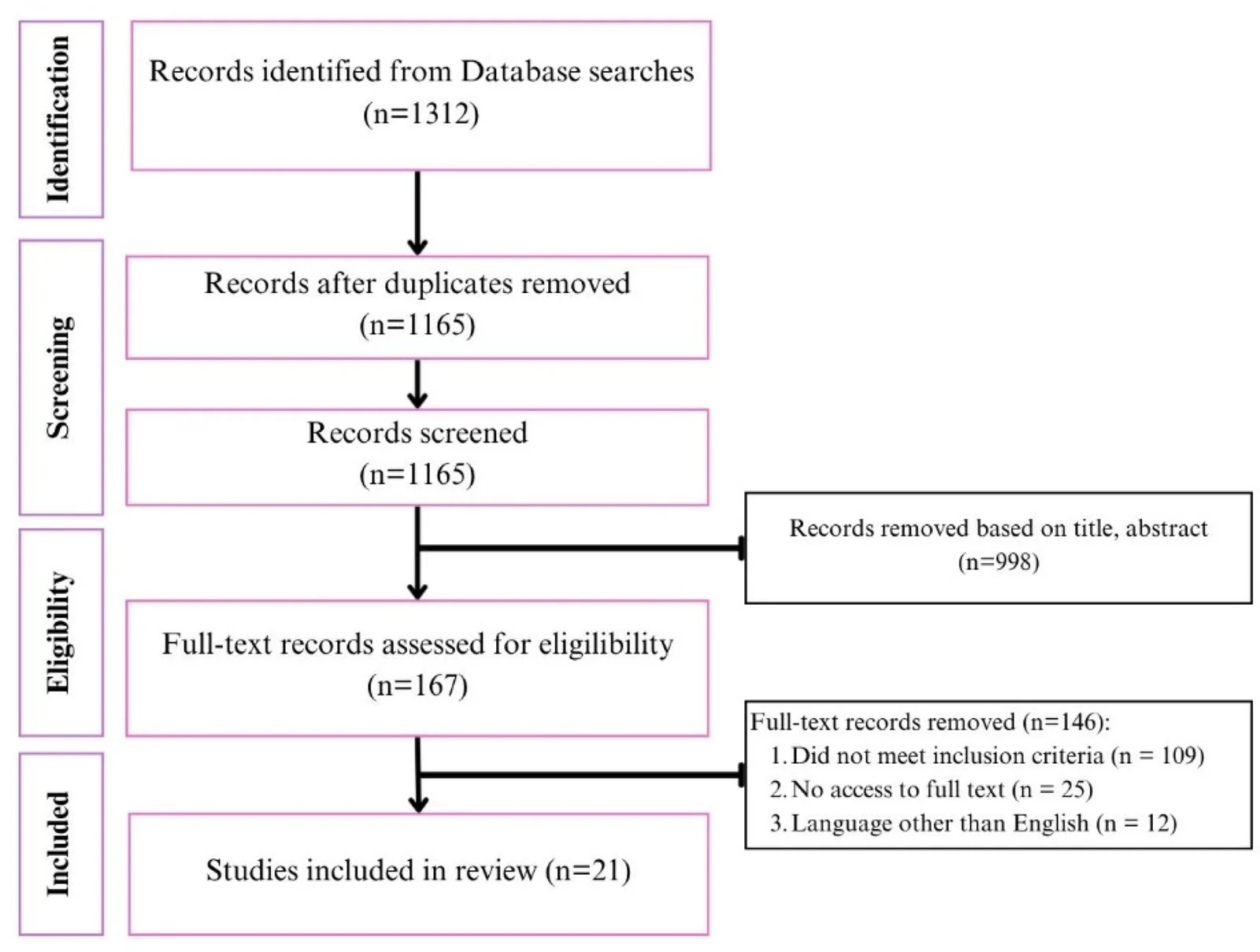
PRISMA flow diagram of the study selection process.

### 3.2 Study characteristics

The 21 studies included in this review comprised a mix of prospective, retrospective, cross-sectional, and observational designs, reflecting a broad range of methodological approaches. The studies were conducted across diverse geographic regions, including Asia, Europe, and Africa, highlighting variability in clinical settings and patient populations.

Sample sizes varied considerably, ranging from small single-center studies with fewer than 50 participants to larger cohorts exceeding 500 patients. Most studies used laparoscopy, often in combination with chromopertubation or hysteroscopy, as the reference standard for assessing tubal patency. The included studies evaluated several diagnostic modalities, primarily HSG, HyCoSy, and HyFoSy. While HSG studies were more heterogeneous in terms of diagnostic performance and reported outcomes, ultrasound-based techniques increasingly incorporated advanced approaches such as power Doppler and foam-based contrast to improve visualization and diagnostic accuracy. A detailed overview of the characteristics and key findings of the included studies is presented in Tables 1, 2.

**Table 1:** Diagnostic performance and agreement findings for HSG in the assessment of tubal-factor infertility (2015–2025).

Study	Country	Design	Patients (n)	Reference standard	Sensitivity (%)	Specificity (%)	Accuracy (%)	PPV (%)	NPV (%)	Agreement/*κ*	Key findings
Ngowa et al. (2015) (13)	Cameroon	Cross-sectional	208	Laparoscopy	51.0–86.8	42.2–90.0	60.9–69.0	40.0–89.3	43.6–90.5	NR	Marked variability; poor detection of adhesions
Gharekhanloo et al. (2017) (14)	Iran	Prospective	115	Laparoscopy	Unilateral: 75.0; bilateral: 75.0	Unilateral: 91.2; bilateral: 97.2	unilateral 89.5; bilateral 96.6	PPV: unilateral 50.0; bilateral 33.0	NPV: unilateral 96.0; bilateral 98.0	NR	High specificity; reliable for occlusion
Chowdhury et al. (2020) (15)	Bangladesh	Prospective	125	Laparoscopic chromopertubation	75.0	50.6	58.4	41.7	81.1	NR	Low specificity; high false-positive rate
Das et al. (2020) (16)	India	Observational	197	Laparoscopic chromopertubation	80.85	74.0	75.63	49.35	92.5	κ = 0.45	Moderate accuracy; high NPV supports screening role
Panda et al. (2021) (17)	India	Prospective	157	Hysterolaparoscopy	100	84.4	88.5	69.4	100	κ = 0.74	Excellent sensitivity; moderate specificity
Popović et al. (2022) (18)	Serbia	Retrospective	63	Laparoscopy	NR	NR	NR	80.8	95.3	Agreement: 77.8%; κ = 0.68	Moderate–good agreement with laparoscopy
Aphinives et al. (2022) (19)	Thailand	Retrospective	515	Hysteroscopy + laparoscopy	89.0	70.0	78.9	72.7	87.5	NR	Moderate accuracy; limited laparoscopy subset
Banotra et al. (2022) (20)	India	Prospective	68	Laparoscopy	91.3	88.9	89.7	NR	NR	NR	Strong overall diagnostic performance
Igbodike et al. (2022) (21)	Nigeria	Prospective	96	Hysterolaparoscopy	right 71.9; left 78.1; bilateral 80.0	right 85.9; left 75.0; bilateral 77.1.	right 77.8; left 76.3; bilateral 78.3	right 71.9; left 69.4; bilateral 71.7.	right 85.9; left 82.6; bilateral 84.2	NR	Moderate diagnostic accuracy
Yildirim et al. (2023) (22)	Turkey	Retrospective	106	Laparoscopy + hysteroscopy	85.0	65.0	80.2	88.3	58.6	NR	High sensitivity but low specificity; supports role of HSG as screening tool; invasive methods needed for confirmation
Sharma et al. (2023) (23)	India	Prospective	105	Laparoscopy	Unilateral: 85.0; bilateral: 81.8	Unilateral: 96.4; bilateral: 98.0	Unilateral block: 94.2; bilateral block: 96.2	Unilateral block: 85.0; bilateral block: 81.8	Unilateral block: 96.0; bilateral block: 98.0	NR	Very high specificity; strong rule-in value
Tabassum et al. (2025) (24)	Bangladesh	Cross-sectional	49	Laparoscopic chromopertubation	91.7	23.1	73.5	76.7	50.0	NR	Very low specificity; poor confirmation ability

HSG, hysterosalpingography; κ, Cohen's kappa coefficient; NPV, negative predictive value; NR, not reported; PPV, positive predictive value.

Diagnostic estimates are presented as reported in the original studies. Direct comparison should be undertaken cautiously because studies differed in patient selection, unit of analysis, definitions of tubal obstruction, and reference standards. Diagnostic accuracy estimates are shown only when reported against a reference standard. Agreement or concordance measures are presented separately and should not be interpreted as sensitivity or specificity.

**Table 2:** Diagnostic performance of hyCoSy and hyFoSy for tubal-factor infertility (2015–2025).

Study	Country	Design	Patients (n)	Method	Contrast medium/formulation	Reference standard or comparator	Sensitivity (%)	Specificity (%)	Accuracy (%)	PPV (%)	NPV (%)	Agreement/concordance/κ	Key findings
Moro et al. (2015) (29)	Italy	Retrospective	126	HyCoSy	Air and sterile saline solution; 15 mL of each instilled through Foley catheter	Laparoscopy with chromopertubation	85.0	93.0	91.0–92.0	71.0–81.0	94.0–97.0	NR	High diagnostic performance in both endometriosis and non-endometriosis patients; selective verification should be considered
Soliman et al. (2015) (32)	Egypt	Prospective	50	Power Doppler/4D HyCoSy	Normal saline/sterile saline	Laparoscopy with chromopertubation	Power Doppler: 94.4; 4D: 70.4	Power Doppler: 100; 4D: 100	Power Doppler: 96.2; 4D: 82.6	Power Doppler: 100; 4D: 100	Power Doppler: 89.2; 4D: 70.4	NR	Power Doppler saline hysterosalpingography showed higher accuracy than 4D saline hysterosalpingography; 40% reported mild abdominal pain; no post-procedural infections
Piccioni et al. (2016) (33)	Italy	Prospective randomized study	37	HyCoSy and HyFoSy	HyFoSy: ExEm-gel foam; HyCoSy: saline solution + air	Laparoscopy with chromopertubation	HyFoSy: 87.5; HyCoSy: 50.0	HyFoSy: 100; HyCoSy: 66.6	HyFoSy: 94.4; HyCoSy: 57.9	NR	NR	Concordanc HyFoSy: 94.4%; HyCoSy: 57.8%	Foam-based sono-HSG showed higher agreement and diagnostic estimates than saline-based HyCoSy, but the sample was small
Ludwin et al. (2017) (30)	Poland	Prospective observational	132	2D air/saline HyCoSy; 2D/3D HyFoSy; 2D/3D-HDF HyFoSy	HyCoSy: air/saline; HyFoSy: ExEm Foam kit	Laparoscopy with chromopertubation	HyCoSy: 93; HyFoSy: 87; HDF-HyFoSy: 94	HyCoSy: 86; HyFoSy: 94; HDF-HyFoSy: 97	HyCoSy: 86.8; HyFoSy: 93.7; HDF-HyFoSy: 96.9	HyCoSy: 30; HyFoSy: 48; HDF-HyFoSy: 71	HyCoSy: 99.5; HyFoSy: 99.0; HDF-HyFoSy: 99.6	κ: HyCoSy 0.40; HyFoSy 0.59; HDF-HyFoSy 0.79	HDF-HyFoSy had the highest PPV and agreement; all methods had high NPV
Krishnamoorthy et al. (2020) (34)	India	Cross-sectional	42	HyCoSy	SonoVue microbubble contrast	Hysterolaparoscopy with chromopertubation	Left tube: 76.5; right tube: 87.5	Left tube: 84.0; right tube: 92.3	Left tube: 81.0; right tube: 90.4	Left tube: 76.5; right tube: 87.5*	Left tube: 84.0; right tube: 92.3*	NR	Variable sensitivity by side; high specificity; supports HyCoSy as a screening method
Ramos et al. (2021) (35)	Spain	Prospective agreement study	106	HyFoSy	ExEm Foam kit/ExEm foam	Previous HSG comparator	NR	NR	NR	NR	NR	Concordance: 72.6%; κ = 0.57	Moderate agreement with HSG; lower reported pain; feasible in an examination room with ultrasound scanner
Sharaf et al. (2022) (31)	Egypt	Prospective	115	HyLiFoSy with power Doppler	Lidocaine gel foam prepared with saline	Laparoscopy with chromopertubation	98.1	90.0	97.4	99.0	81.8	Concordance: 94.8% per patient; 97.4% per tube	High diagnostic accuracy; office-based procedure; mild pain in 79% and moderate pain in 21%; no other procedure-related complications
Le et al. (2024) (36)	Vietnam	Prospective diagnostic study	447	2D HyFoSy	ExEm Foam kit/ExEm gel, GynaecologIQ	Same-day laparoscopy with dye chromotubation	75.0	70.0	NR	76.0	68.0	NR	Well tolerated; 42.8% reported no pain and no adverse events occurred
Wu et al. (2025) (37)	China	Retrospective	76	4D-HyCoSy	Perfluoropropane human albumin microspheres diluted in physiological saline	Laparoscopy with dye test	60.0–78.26	67.27–74.14	70.51	NR	NR	Concordance: 70.5%; κ = 0.546	Comparable diagnostic performance and lower reported pain

HDF, high-definition flow; HSG, hysterosalpingography; HyCoSy, hysterosalpingo-contrast sonography; HyFoSy, hysterosalpingo-foam sonography; κ, Cohen's kappa coefficient; LR+, positive likelihood ratio; NPV, negative predictive value; NR, not reported; PPV, positive predictive value. *PPV and NPV were recalculated only when sufficient 2 × 2 data were reported in the original article. NR indicates not reported or not clearly specified.

Diagnostic estimates are presented as reported in the original studies. PPV and NPV are influenced by the prevalence of tubal obstruction in the study population. Agreement with another non-invasive test, such as HSG, is not equivalent to diagnostic sensitivity or specificity against laparoscopy with chromopertubation. Diagnostic accuracy estimates are shown only when reported against a reference standard. Agreement or concordance measures are presented separately and should not be interpreted as sensitivity or specificity.

### 3.3 Methodological quality and risk of bias

The methodological quality of the included studies was assessed using appraisal tools selected according to study design and purpose. Diagnostic-accuracy studies comparing an index test with a defined reference standard were evaluated using the QUADAS-2 tool. Studies primarily assessing agreement or concordance between diagnostic methods without an independent reference standard were evaluated using the Joanna Briggs Institute Critical Appraisal Checklist for Analytical Cross-Sectional Studies.

Among the studies assessed using QUADAS-2, the reference-standard domain generally demonstrated the lowest risk of bias because most studies used laparoscopy with chromopertubation or combined hysterolaparoscopy with dye testing. However, methodological concerns were identified in the patient-selection, index-test, and flow-and-timing domains. High risk of bias in patient selection or flow and timing was mainly related to selective or partial verification, as some studies performed the reference standard only in women with abnormal index-test findings, persistent infertility, or specific clinical indications. These concerns affected the interpretation of diagnostic estimates. Studies in which laparoscopy with chromopertubation was performed only in selected patients were interpreted cautiously, because partial verification may distort estimates of sensitivity, specificity, PPV, and NPV. Therefore, findings from studies with high selection or flow-and-timing concerns were not treated as equivalent to studies in which all or most participants received the same reference standard. In several studies, the risk of bias in the index-test domain was judged unclear because blinding of index-test interpretation to the reference-standard results or prespecification of diagnostic thresholds was not adequately reported.

Popović et al. and Ramos et al. were assessed separately using the JBI checklist because their primary analyses focused on agreement or concordance rather than diagnostic accuracy against an independent reference standard. Both studies adequately described their populations, settings, and statistical methods; however, limitations were identified in relation to the assessment and management of potential confounding factors. In Popović et al., additional concerns arose from insufficient reporting of standardized diagnostic criteria and blinding.

A summary of the QUADAS-2 risk-of-bias assessment is presented in Figure 2. Detailed study-level QUADAS-2 judgments are provided in Supplementary Table S2, while the JBI assessments of the agreement and concordance studies are presented separately in Supplementary Table S3.

**Figure 2:**
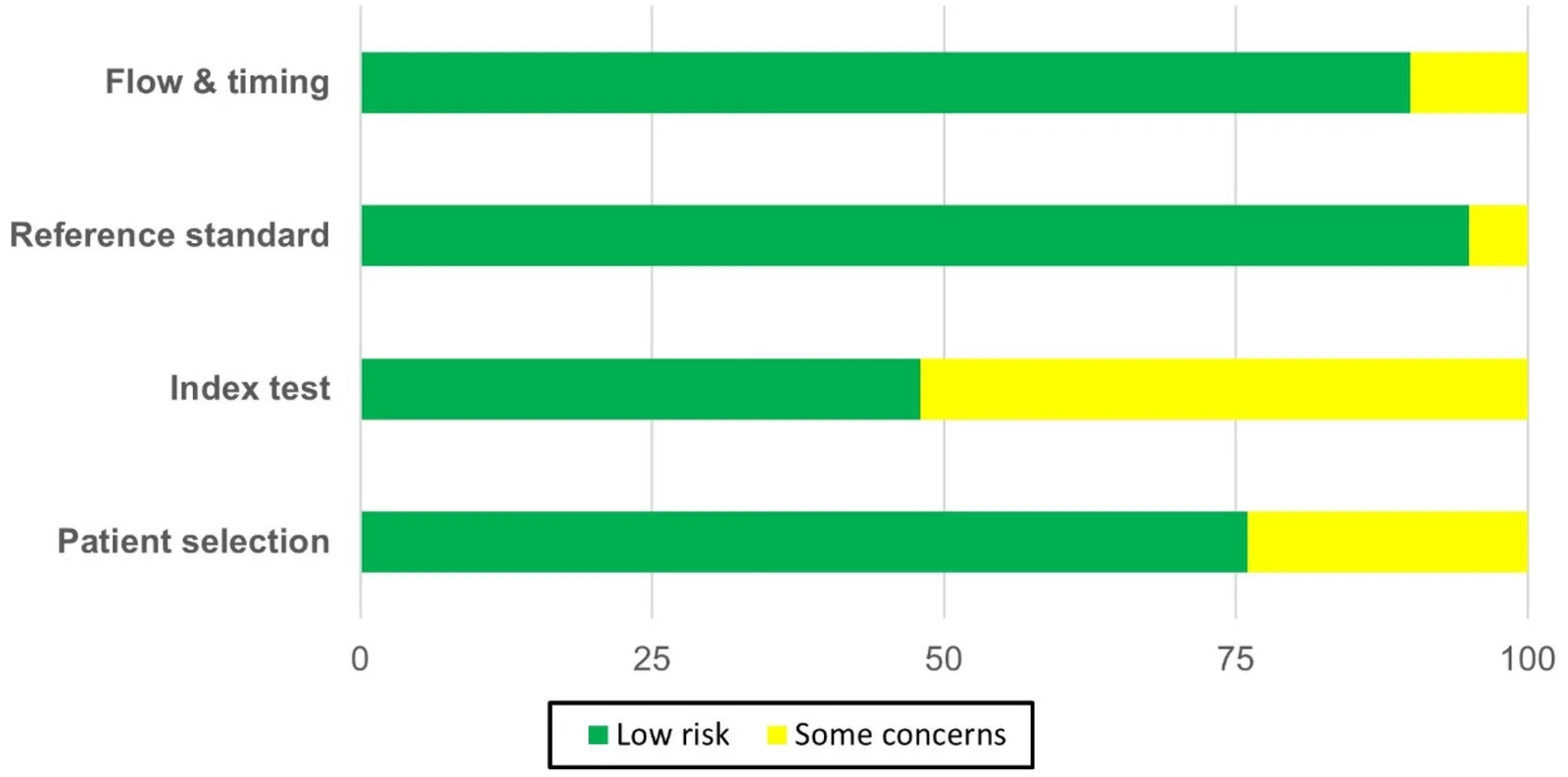
Summary of risk of bias across included studies according to QUADAS-2 domains.

### 3.4 Diagnostic performance of methods

The diagnostic performance of methods used to assess tubal patency and morphology varied across studies. This variation likely reflected differences in study design, patient populations, prevalence of tubal pathology, reference standards, diagnostic thresholds, operator expertise, imaging protocols, and units of analysis. Therefore, reported sensitivity, specificity, predictive values, and agreement measures should be interpreted cautiously and should not be considered directly interchangeable across studies. The following sections summarize the key findings for the most commonly used diagnostic approaches, including HSG and HyCoSy and its modifications, with laparoscopy serving as the reference standard.

#### 3.4.1 Hysterosalpingography (HSG)

Hysterosalpingography remains a widely used first-line modality for the assessment of tubal patency in women undergoing infertility evaluation (Table 1). Across the included studies, HSG demonstrated generally high sensitivity, typically ranging from approximately 75% to over 90%, indicating a strong ability to detect tubal abnormalities (13, 14). In contrast, specificity varied considerably, with reported values ranging from as low as 23% to nearly 98% (15–23). This marked variability reflects differences in study design, patient populations, and technical factors. Agreement with laparoscopy was reported as moderate to good, with concordance rates around 75%–80% (18, 21), supporting the role of HSG as a screening rather than definitive diagnostic tool.

The variability in diagnostic performance may partly reflect differences in study populations, disease prevalence, procedural protocols, contrast administration, diagnostic thresholds, interpretation criteria, and the definition of tubal abnormality. Several studies reported high negative predictive values, suggesting that HSG may be useful for excluding clinically significant tubal obstruction when findings are normal (16, 20).

From a practical perspective, HSG is widely available, relatively inexpensive, and suitable for outpatient use (25, 26). Its principal modality-specific disadvantages include exposure to ionizing radiation, the use of iodinated contrast media, and procedural discomfort during cervical instrumentation and contrast injection (27, 28).

In general, HSG remains a valuable initial screening tool in the evaluation of tubal infertility. Nevertheless, its limited specificity and inability to reliably detect extra-tubal disease highlight the need for complementary diagnostic approaches, particularly in cases with inconclusive or discordant findings.

#### 3.4.2 Hycosy and HyFoSy

Across the included studies, HyCoSy and HyFoSy demonstrated generally high diagnostic performance for the assessment of tubal patency, with notable variability depending on technique and study design (29–31) (Table 2). Early studies evaluating conventional HyCoSy reported high sensitivity and specificity, typically exceeding 85%, particularly when enhanced techniques such as power Doppler were used (32). However, variability in sensitivity was observed in some cohorts, with reported values ranging from as low as 40% to over 90%, suggesting that diagnostic performance may be influenced by operator experience, patient selection, and technical factors (34).

Several comparative studies reported more favorable diagnostic estimates for HyFoSy than for conventional HyCoSy (30, 33). Foam-based contrast agents may improve visualization of tubal flow and reduce equivocal or false-positive findings (30, 32). In particular, studies directly comparing both techniques suggested that HyCoSy may provide high negative predictive value and may be useful as an initial screening tool, whereas HyFoSy showed favorable diagnostic performance in selected studies, particularly for evaluating suspected tubal obstruction (7, 10). In addition to its diagnostic performance, HyFoSy has been reported to have favorable patient tolerability and lower procedure-related pain compared with HSG in some single-center and multicenter studies (38, 39).

When compared with laparoscopy with chromopertubation, HyFoSy showed favorable sensitivity and specificity in some studies (31). However, diagnostic estimates were not uniform, and agreement-based studies using HSG as a comparator reported more moderate concordance, reflecting agreement between two non-invasive methods rather than diagnostic accuracy against an independent reference standard (35, 37).

From a clinical perspective, both HyCoSy and HyFoSy offer several practical advantages. They avoid ionizing radiation and can generally be performed as outpatient procedures using transvaginal ultrasound equipment. They may be undertaken by appropriately trained clinicians and do not necessarily require fluoroscopy or the involvement of a specialized radiologist. Ultrasound-based assessment also permits simultaneous evaluation of the uterus, ovaries, and other visible pelvic structures during the infertility workup (40). Implementation nevertheless depends on appropriate operator training, equipment availability, local regulations, and institutional practice.

Patient-tolerability findings were particularly favorable for foam-based techniques. Several studies reported lower procedural pain or improved tolerability compared with HSG, although pain-measurement instruments and reporting methods differed across studies (38, 39, 41, 42).

Overall, the available evidence supports HyCoSy and HyFoSy as useful and patient-friendly methods for tubal-patency assessment. HyFoSy appears promising because of its favorable diagnostic and tolerability profile in selected studies, but further standardized direct-comparison studies are needed before it can be considered consistently superior to HyCoSy or HSG.

### 3.5 Safety, patient tolerability, and practical applicability

Safety, procedural pain, and practical applicability were reported inconsistently across the included studies. HSG was generally described as an accessible outpatient procedure; however, it involves exposure to ionizing radiation and iodinated contrast media and may cause discomfort during cervical instrumentation and contrast injection. Several studies also noted that false-positive occlusion may occur because of transient tubal spasm or technical factors.

Ultrasound-based methods avoid ionizing radiation and can generally be performed in an outpatient setting using transvaginal ultrasound equipment. HyCoSy was described as a simple and minimally invasive procedure that usually did not require hospitalization or anesthesia. Nevertheless, its performance was operator-dependent and influenced by the ultrasound technique and contrast medium used.

Patient-tolerability outcomes were reported most frequently for HyFoSy. HyFoSy was associated with less pain than previous HSG, with 59.1% of participants reporting no pain during HyFoSy and only 6.6% describing it as more painful than HSG. The procedure was also considered feasible in a standard examination room equipped with an ultrasound scanner (35). In another study, 42.8% of women reported no pain during HyFoSy, and no adverse events were recorded (36). All participants experienced some degree of procedural pain during HyLiFoSy with power Doppler; however, pain was mild in 79% and moderate in 21%, with no severe pain reported. The median procedure duration was 11 min, and the technique was described as an office-based procedure that could be performed using conventional two-dimensional transvaginal ultrasound equipment (31).

Foam-based sonography was also reported to provide improved visualization and better tolerability than saline-based sonography, although detailed pain scores were not presented (33). Overall, the available evidence suggests that HyCoSy and HyFoSy may offer practical advantages in terms of outpatient delivery, avoidance of radiation, and patient tolerability. However, comparisons should be interpreted cautiously because pain scales, analgesic protocols, operators, contrast formulations, and reporting methods varied across studies.

### 3.6 Comparative analysis of diagnostic methods

The comparative analysis of available diagnostic methods highlights not only differences in performance metrics but, more importantly, their distinct roles within the clinical evaluation of tubal factor infertility. Rather than representing a validated clinical algorithm, these findings suggest an evidence-informed framework in which diagnostic methods may be selected according to clinical context, availability, patient preference, and the need for confirmation.

Across the included studies, a consistent pattern emerges in which HSG and conventional HyCoSy function primarily as initial screening tools. Their accessibility, relatively low cost, and ability to exclude significant tubal pathology make them suitable for first-line evaluation (16, 30, 34). However, their limited specificity and susceptibility to false-positive findings reduce their reliability when confirming tubal occlusion, particularly in cases where clinical suspicion remains high (15, 24, 33). These observations are supported by recent meta-analyses demonstrating variability in specificity and the diagnostic limitations of conventional techniques (7, 10).

HyFoSy may offer a favorable balance between diagnostic performance and procedural tolerability in selected settings. Foam-based contrast may improve visualization and reduce inconclusive findings, but the strength of evidence is limited by heterogeneous study designs, variable reference standards, and differences in operator expertise and contrast formulations (30, 31, 33). Recent systematic reviews have reported favorable findings for foam-based contrast techniques compared with conventional HyCoSy, particularly in relation to specificity and agreement with laparoscopy; however, these conclusions should be interpreted cautiously because of heterogeneity across the available studies (7, 38).

Beyond diagnostic performance, the choice of modality is also influenced by patient-centered and practical considerations. While HSG remains widely available, its association with higher discomfort and radiation exposure may limit its acceptability (38, 43). Ultrasound-based techniques, including HyCoSy and HyFoSy, offer a more favorable patient experience and allow simultaneous evaluation of pelvic structures, supporting a more comprehensive and less invasive diagnostic approach (39). These findings are supported by recent reviews emphasizing improved tolerability and patient preference for ultrasound-based methods (35, 38).

Despite these advantages, none of the non-invasive modalities fully replace laparoscopy, which remains the reference standard due to its ability to directly visualize both tubal patency and extra-tubal pathology (38). However, given its invasive nature, laparoscopy is best reserved for cases where non-invasive methods yield inconclusive results or when additional pelvic pathology is suspected.

Taken together, these findings support a structured, stepwise approach to tubal evaluation, in which less invasive methods are used for initial assessment, followed by more accurate or invasive techniques when clinically indicated. This strategy allows optimization of diagnostic accuracy while minimizing patient burden and procedural risk.

## 4 Discussion

This systematic review provides an overview of the diagnostic methods currently used to assess tubal-factor infertility, with particular attention to diagnostic performance, clinical applicability, patient tolerability, and methodological limitations. Overall, the findings indicate that no single diagnostic method is optimal in every clinical setting. HSG, HyCoSy, and HyFoSy should therefore be viewed as complementary rather than universally interchangeable techniques. The main implication of these findings is that diagnostic estimates should not be interpreted as a simple ranking of tests. The observed variation in sensitivity, specificity, predictive values, and agreement reflects differences in patient populations, prevalence of tubal pathology, reference standards, diagnostic thresholds, operator expertise, and study design. Therefore, a method that performs well in a specialist center or in a selected population may not show the same performance in routine clinical practice. This is particularly important for ultrasound-based techniques, where technical quality, contrast formulation, and operator experience may influence visualization of tubal flow.

HSG remains widely used as an initial diagnostic test because of its availability, familiarity, and relatively low cost. Across the included studies, however, its diagnostic performance varied substantially. Although sensitivity was frequently high, specificity ranged widely, indicating that an abnormal HSG result may not always represent true tubal occlusion. These findings support its use primarily as an initial screening method rather than as a definitive confirmatory test.

HyCoSy offers the advantages of avoiding ionizing radiation and allowing simultaneous ultrasound assessment of the uterus, ovaries, and visible pelvic structures. Nevertheless, its reported sensitivity and specificity were inconsistent. This variation may be related to operator experience, ultrasound equipment, contrast materials, imaging protocols, and the criteria used to define tubal patency (6). Therefore, findings obtained in experienced specialist centers may not be directly generalizable to all clinical environments.

Several included studies reported favorable diagnostic estimates for HyFoSy, including high specificity, improved visualization, and better patient tolerability (31, 38, 39). However, these findings should be interpreted cautiously. The evidence was derived from a limited number of heterogeneous studies, some of which had relatively small samples or single-center designs. Moreover, not all studies used laparoscopy with chromopertubation as the reference standard. For example, studies comparing HyFoSy with HSG primarily assessed agreement between two non-invasive methods rather than diagnostic accuracy against a definitive reference standard (35). Consequently, the available evidence does not establish that HyFoSy is consistently superior to HSG or HyCoSy across all populations and clinical settings. Thus, HyFoSy should be interpreted as a promising patient-friendly option rather than as a universally validated replacement for existing diagnostic methods.

All non-invasive imaging methods for tubal-patency assessment share several limitations. They evaluate tubal patency indirectly and may produce false-positive findings, particularly apparent proximal occlusion caused by transient tubal spasm, mucus plugs, technical difficulties during contrast administration, or differences in image interpretation (4, 7, 10). Their performance may also be influenced by operator experience, image quality, contrast medium, procedural technique, and interpretation criteria (6). In addition, these methods have limited ability to identify extra-tubal pelvic abnormalities, including endometriosis and peritubal adhesions. Therefore, laparoscopy with chromopertubation remains clinically relevant when associated pelvic pathology is suspected, non-invasive findings are inconclusive, or test results are inconsistent with the clinical presentation (43).

The apparent advantages of HyFoSy may also be influenced by differences in disease prevalence, patient selection, operator expertise, foam formulation, and the unit of analysis used. Some studies reported outcomes per patient, whereas others evaluated individual fallopian tubes. These approaches are not directly equivalent and may produce different estimates of sensitivity, specificity, and agreement. In addition, positive predictive value and negative predictive value depend on the prevalence of tubal obstruction in the study population and cannot be interpreted as fixed characteristics of the test.

From a clinical perspective, these findings support an evidence-informed and individualized diagnostic framework rather than a directly validated diagnostic algorithm. In women with low clinical suspicion of extra-tubal pelvic disease, less invasive methods such as HSG, HyCoSy, or HyFoSy may be considered for initial assessment, depending on local availability, operator expertise, patient preference, cost, contraindications, and the need to avoid radiation. However, abnormal or discordant results from non-invasive tests should be interpreted cautiously, particularly when the study evidence is affected by selection or verification bias. In such cases, confirmation with another diagnostic method may be appropriate. Laparoscopy with chromopertubation remains clinically relevant when endometriosis, pelvic adhesions, hydrosalpinx, prior pelvic infection, or another surgically treatable pelvic pathology is suspected.

Procedure-related pain and patient tolerability are also important considerations when selecting a tubal-patency test. HSG may cause discomfort during cervical instrumentation and the injection of iodinated contrast, whereas ultrasound-based techniques avoid radiation and were generally well tolerated. Several included studies reported lower pain or favorable tolerability with HyFoSy compared with HSG, including substantial proportions of women reporting no or only mild pain (36–41). However, direct comparison was limited by differences in pain scales, analgesic protocols, operator experience, contrast formulations, and timing of pain assessment. Therefore, although HyFoSy may offer an additional patient-centered advantage, the available tolerability findings should be interpreted cautiously.

Laparoscopy with chromopertubation remains an important reference method because it allows direct assessment of tubal patency and simultaneous visualization of extra-tubal pelvic pathology (43). Nevertheless, its invasiveness, cost, need for anesthesia, and procedural risks make it unsuitable as a routine first-line investigation for all women. It is more appropriately reserved for selected patients, particularly when endometriosis, pelvic adhesions, or another surgically treatable abnormality is suspected.

The strengths of this review include the structured comparison of several commonly used diagnostic modalities and the consideration of both diagnostic outcomes and patient-centered factors, including pain, tolerability, radiation exposure, and invasiveness. The inclusion of recent studies also provides an overview of contemporary diagnostic practice.

Several limitations should nevertheless be acknowledged. First, the included studies differed substantially in design, sample size, patient characteristics, prevalence of tubal pathology, diagnostic protocols, contrast agents, diagnostic thresholds, operator experience, units of analysis, and reference standards. Several studies had relatively small sample sizes or single-center designs, which may limit the generalizability of their findings. These differences also limited direct comparison between studies and prevented quantitative pooling. For these reasons, quantitative pooling could have produced misleading summary estimates and was not undertaken. Second, diagnostic outcomes were inconsistently reported. Some studies provided sensitivity and specificity, whereas others reported only accuracy, predictive values, likelihood ratios, concordance, or Cohen's kappa. Agreement between two tests should not be interpreted as equivalent to diagnostic accuracy against an accepted reference standard. Third, selection and verification bias were important methodological concerns. In some studies, laparoscopy with chromopertubation was performed only in selected patients, such as those with abnormal index-test findings, persistent infertility, or clinical indications for invasive evaluation. This may have influenced estimates of sensitivity, specificity, PPV, and NPV. In addition, inadequate reporting of blinding of index-test interpretation may have introduced review bias. Fourth, the operator-dependent nature of ultrasound-based methods may limit reproducibility outside specialist centers. Fifth, restricting the review to English-language publications may have introduced language bias and excluded relevant studies published in other languages. The exclusion of articles for which full texts were unavailable may also have introduced availability bias.

Future studies should use prospective, multicenter designs and standardized diagnostic protocols. They should clearly define the unit of analysis, criteria for tubal occlusion, operator training, and reference standard. Diagnostic studies should report sensitivity, specificity, accuracy, positive predictive value, negative predictive value, likelihood ratios, and agreement statistics consistently. Direct head-to-head comparisons of HSG, HyCoSy, and HyFoSy in the same patient population would provide stronger evidence than indirect comparisons across heterogeneous studies.

In conclusion, HSG, HyCoSy, and HyFoSy are complementary options within the diagnostic evaluation of tubal-factor infertility. HyFoSy appears promising, particularly in terms of tolerability and favorable diagnostic performance in selected studies, but the available evidence remains insufficient to establish its universal superiority. Selection of the diagnostic method should be based on clinical context, suspected extra-tubal pathology, local expertise, availability, and patient preferences.

## Data Availability

The original contributions presented in the study are included in the article/Supplementary Material, further inquiries can be directed to the corresponding author.
