# Supplementary material for: Diagnostic approaches for tubal factor infertility: a systematic review of HSG, HyCoSy, HyFoSy, and laparoscopy
Source: Front Reprod Health. 2026 Oct 7;8:1861471. doi: 10.3389/frph.2026.1861471 (PMC13683700; doi:10.3389/frph.2026.1861471)
Supplement: Supplementary file 1 [file Datasheet1.docx]

**SUPPLEMENTARY MATERIALS**

**Supplementary Table 1.** Database-specific search strategies

| **Database** | **Search strategy** |
| --- | --- |
| **PubMed/MEDLINE** | (("Infertility, Female"[Mesh] OR infertil*[tiab] OR subfertil*[tiab]) AND ("Fallopian Tubes"[Mesh] OR "tubal factor"[tiab] OR "tubal infertility"[tiab] OR "fallopian tube obstruction"[tiab] OR "fallopian tube occlusion"[tiab] OR "tubal obstruction"[tiab] OR "tubal occlusion"[tiab] OR "tubal patency"[tiab] OR "fallopian tube patency"[tiab]) AND ("Hysterosalpingography"[Mesh] OR hysterosalpingograph*[tiab] OR HSG[tiab] OR "hysterosalpingo-contrast sonography"[tiab] OR "hysterosalpingo contrast sonography"[tiab] OR HyCoSy[tiab] OR "hysterosalpingo-foam sonography"[tiab] OR "hysterosalpingo foam sonography"[tiab] OR HyFoSy[tiab] OR "Laparoscopy"[Mesh] OR laparoscop*[tiab] OR chromopertubation[tiab] OR chromotubation[tiab] OR "dye test"[tiab]) AND ("Sensitivity and Specificity"[Mesh] OR "diagnostic accuracy"[tiab] OR sensitivity[tiab] OR specificity[tiab] OR accuracy[tiab] OR PPV[tiab] OR NPV[tiab] OR "predictive value"[tiab] OR "likelihood ratio"[tiab] OR concordance[tiab] OR agreement[tiab] OR kappa[tiab])) Filters: English, humans, publication date from 1 January 2015 to 31 March 2026. |
| **Embase** | ('female infertility'/exp OR infertil*:ti,ab,kw OR subfertil*:ti,ab,kw) AND ('fallopian tube'/exp OR 'tubal factor':ti,ab,kw OR 'tubal infertility':ti,ab,kw OR 'fallopian tube obstruction':ti,ab,kw OR 'fallopian tube occlusion':ti,ab,kw OR 'tubal obstruction':ti,ab,kw OR 'tubal occlusion':ti,ab,kw OR 'tubal patency':ti,ab,kw OR 'fallopian tube patency':ti,ab,kw) AND ('hysterosalpingography'/exp OR hysterosalpingograph*:ti,ab,kw OR hsg:ti,ab,kw OR 'hysterosalpingo contrast sonography':ti,ab,kw OR 'hysterosalpingo-contrast sonography':ti,ab,kw OR hycosy:ti,ab,kw OR 'hysterosalpingo foam sonography':ti,ab,kw OR 'hysterosalpingo-foam sonography':ti,ab,kw OR hyfosy:ti,ab,kw OR 'laparoscopy'/exp OR laparoscop*:ti,ab,kw OR chromopertubation:ti,ab,kw OR chromotubation:ti,ab,kw OR 'dye test':ti,ab,kw) AND ('diagnostic accuracy'/exp OR sensitivity:ti,ab,kw OR specificity:ti,ab,kw OR accuracy:ti,ab,kw OR ppv:ti,ab,kw OR npv:ti,ab,kw OR 'predictive value':ti,ab,kw OR 'likelihood ratio':ti,ab,kw OR concordance:ti,ab,kw OR agreement:ti,ab,kw OR kappa:ti,ab,kw) Limits: English language, human studies, publication year 2015–2026. |
| **Cochrane Library / CENTRAL** | ([mh "Infertility, Female"] OR infertil*:ti,ab,kw OR subfertil*:ti,ab,kw) AND ([mh "Fallopian Tubes"] OR "tubal factor":ti,ab,kw OR "tubal infertility":ti,ab,kw OR "fallopian tube obstruction":ti,ab,kw OR "fallopian tube occlusion":ti,ab,kw OR "tubal obstruction":ti,ab,kw OR "tubal occlusion":ti,ab,kw OR "tubal patency":ti,ab,kw OR "fallopian tube patency":ti,ab,kw) AND ([mh Hysterosalpingography] OR hysterosalpingograph*:ti,ab,kw OR HSG:ti,ab,kw OR HyCoSy:ti,ab,kw OR HyFoSy:ti,ab,kw OR "hysterosalpingo-contrast sonography":ti,ab,kw OR "hysterosalpingo-foam sonography":ti,ab,kw OR [mh Laparoscopy] OR laparoscop*:ti,ab,kw OR chromopertubation:ti,ab,kw OR chromotubation:ti,ab,kw OR "dye test":ti,ab,kw) AND ("diagnostic accuracy":ti,ab,kw OR sensitivity:ti,ab,kw OR specificity:ti,ab,kw OR accuracy:ti,ab,kw OR PPV:ti,ab,kw OR NPV:ti,ab,kw OR "predictive value":ti,ab,kw OR "likelihood ratio":ti,ab,kw OR concordance:ti,ab,kw OR agreement:ti,ab,kw OR kappa:ti,ab,kw) Limits: publication date from January 2015 to March 2026; English language where filter was available. |
| **Web of Science** | TS=((infertil* OR subfertil*) AND ("tubal factor" OR "tubal infertility" OR "fallopian tube obstruction" OR "fallopian tube occlusion" OR "tubal obstruction" OR "tubal occlusion" OR "tubal patency" OR "fallopian tube patency") AND (hysterosalpingograph* OR HSG OR HyCoSy OR HyFoSy OR "hysterosalpingo-contrast sonography" OR "hysterosalpingo contrast sonography" OR "hysterosalpingo-foam sonography" OR "hysterosalpingo foam sonography" OR laparoscopy OR laparoscop* OR chromopertubation OR chromotubation OR "dye test") AND ("diagnostic accuracy" OR sensitivity OR specificity OR accuracy OR PPV OR NPV OR "predictive value*" OR "likelihood ratio*" OR concordance OR agreement OR kappa)) Limits: English language; publication years 2015–2026. |

**Supplementary Figure 1.** Risk-of-bias assessment of included studies using QUADAS-2 domains

| **Study** | **Patient selection** | **Index test** | **Reference standard** | **Flow and timing** |
| --- | --- | --- | --- | --- |
| Ngowa et al. (2015) | High | Unclear | Low | Unclear |
| Moro et al. (2015) | High | Low | Low | High |
| Soliman et al. (2015) | Low | Low | Low | Low |
| Piccioni et al. (2016) | Low | Low | Low | Low |
| Ludwin et al. (2017) | Low | Low | Low | Low |
| Gharekhanloo et al. (2017) | High | Unclear | Low | Unclear |
| Chowdhury et al. (2020) | Low | Unclear | Low | Low |
| Das et al. (2020) | High | Unclear | Low | High |
| Krishnamoorthy et al. (2020) | Unclear | Unclear | Low | Low |
| Panda et al. (2021) | High | Unclear | Low | High |
| Aphinives et al. (2022) | High | Unclear | Low | High |
| Banotra et al. (2022) | Low | Unclear | Low | Low |
| Igbodike et al. (2022) | Low | Low | Low | Low |
| Sharaf et al. (2022) | Low | Low | Low | Low |
| Yildirim et al. (2023) | High | Unclear | Low | High |
| Sharma et al. (2023) | Low | Unclear | Low | Low |
| Le et al. (2024) | Low | Low | Low | Low |
| Tabassum et al. (2025) | Low | Unclear | Low | Unclear |
| Wu et al. (2025) | Unclear | Unclear | Low | Low |

Supplementary Table 3. Critical appraisal of agreement and concordance studies using the JBI Analytical Cross-Sectional Studies Checklist

| **Study** | **1. Inclusion criteria clearly defined** | **2. Participants and setting described in detail** | **3. Diagnostic methods measured validly and reliably** | **4. Objective criteria used to define the condition/outcome** | **5. Confounding factors identified** | **6. Strategies stated to address confounding** | **7. Outcomes measured validly and reliably** | **8. Appropriate statistical analysis used** | **Overall appraisal** |
| --- | --- | --- | --- | --- | --- | --- | --- | --- | --- |
| Popović et al. (2022) | Yes | Yes | Unclear | Unclear | No | No | Unclear | Yes | Include with methodological limitations |
| Ramos et al. (2021) | Yes | Yes | Yes | Yes | No | No | Yes | Yes | Include with methodological limitations |

**Response options:** Yes, criterion adequately addressed; No, criterion not addressed; Unclear, insufficient information reported to permit a judgment.
